# A Two-Step Nanofiltration Process for the Production of Phenolic-Rich Fractions from Artichoke Aqueous Extracts

**DOI:** 10.3390/ijms16048968

**Published:** 2015-04-22

**Authors:** Alfredo Cassano, Carmela Conidi, René Ruby Figueroa, Roberto Castro Muñoz

**Affiliations:** 1 Institute on Membrane Technology, ITM-CNR, c/o University of Calabria, via Pietro Bucci, 17/C, 87036 Rende (CS), Italy; E-Mails: c.conidi@itm.cnr.it (C.C.); r.ruby@itm.cnr.it (R.R.F.); 2 Instituto Politécnico Nacional, Unidad Profesional Interdisciplinaria de Biotecnologia, Av. Acueducto, 5/n, Col. Barrio La Laguna Ticomán, C.P. 07340, Mexico; E-Mail: soruyocm@hotmail.com

**Keywords:** artichoke, phenolic compounds, nanofiltration, antioxidants

## Abstract

Commercial nanofiltration (NF) membranes in spiral-wound configuration (NP030 from Microdyn Nadir and Desal DK from GE Water & Process Technologies) were used in a sequential design in order to produce a separated fraction of phenolic and sugar compounds from an aqueous artichoke extract. For both membranes, the effect of transmembrane pressure (TMP) on the permeation flux was evaluated. In optimized conditions of TMP, the NP030 membrane exhibited high rejections of apigenin, cynarin and chlorogenic acid (higher than 85%); on the other hand, very low rejections of fructose, glucose and sucrose (lower than 4%) were measured. Starting from an extract with a total antioxidant activity (TAA) of 5.28 mM trolox a retentate fraction with a TAA of 47.75 mM trolox was obtained. The NF permeate from the NP030 membrane was processed with the Desal DK membrane in optimized conditions of TMP producing a permeate stream free of phenolic and sugar compounds. Accordingly, as most part of phenolic compounds was removed in the first NF step, the concentration of sugar compounds in the NF retentate had much higher results than that of phenolic compounds.

## 1. Introduction

In recent years, the recovery of antioxidant compounds from natural sources is a great focus of interest due to their potential use as natural ingredients in food, pharmaceutical and cosmetic formulations or as substitutes of synthetic products in the food industry [1].

The artichoke is a native plant of the Mediterranean area, where it is traditionally cultivated and commonly consumed as a vegetable, playing an important role in human nutrition [2]. In comparison with other vegetables, artichokes are low in saturated fat and cholesterol, while being a rich source in fiber, vitamins (including vitamin C, thiamin, riboflavin, niacin, folate, vitamin B-6, B-12, A, E, D and K) minerals (potassium, sodium and phosphorus), sugars and polyphenols [3].

Different studies have demonstrated that artichoke is a rich source of health-promoting compounds and its biochemical components have been extensively characterized [4,5].

Phenolic compounds such as chlorogenic acid, mono- and di-caffeoylquinic acids as well as apigenin, luteolin and the carbohydrate inulin, have been reported to be the major bioactive components of the artichoke and correlated to its antiallergenic, anti-inflammatory, cardio-protective, anti-thrombotic, vaso-dilatatory and hepatoprotective activity [6,7]. Many of these properties are due to antioxidant activity of phenolic compounds, involving various mechanisms such as free radical scavenging, electron or hydrogen atom donation, or metal cation chelation [8,9,10,11]. The nutritional and pharmacological properties of the artichoke have greatly increased the demand of artichoke worldwide and thus its production [12].

Apart from the edible parts of artichoke, the agro-industrial by-products such as leaves, external bracts and stems represent about the 80% of the plant biomass and could be a promising and cheap source of health-promoting phenolic compounds and inulin [4,13].

These by-products are suitable for ensiling, with pleasant smell, good silage characteristics, crude protein content 88 g/kg dry matter and fiber content 509 g/kg dry matter [14]. An alternative approach is their use as sources of natural antioxidant compounds, mainly phenolic compounds, which, in some cases, have activities comparable to those of synthetic antioxidants. For this purpose, Llorach *et al.* [15] evaluated the performance of two protocols, with possible industrial applicability, based on the use of methanol and water extraction to extract phenolic compounds from raw artichoke, blanched (thermally treated) artichoke, and artichoke blanching waters which represent the main discarded materials of the processing artichoke industry.

The optimization of the extraction of bioactive compounds from by-products was intensively studied and a number of methods were proposed including distillation, coagulation and precipitation of impurities, evaporation, chemical extraction, solid phase extraction and extraction by ultrasound [16]. However, the thermal processes (*i.e.*, evaporation) cause a degradation of the target compounds and uncontrolled generation of Maillard by-products. The extraction with solvents is time consuming and requires large amounts of solvent [17,18].

In recent years, more environmentally friendly techniques have been investigated and used for the separation, purification and concentration of bioactive compounds allowing to reduce extraction time and solvent consumption as well as to increase bioactive compounds yield [19].

Membrane operations are recognized as powerful tools for the purification and concentration of various solutions (e.g., juices, extracts, whey) and the separation of valuable compounds from by-products of the agro-food industry [20].

The basic properties of membrane operations make them competitive with conventional methodologies: they do not involve phase changes, chemical additives and heat treatment, they are modular and easy to scale-up, and are characterized by unlimited selectivity of separation, thereby enabling a more rational utilization of raw materials and recovery and reuse of by-products. In addition, they respond efficiently to the requirements of so-called “process intensification”, allowing drastic improvements in manufacturing and processing, substantially decreasing the equipment-size/production-capacity ratio, energy consumption, and/or waste production [21,22].

Pressure-driven membrane operations such as microfiltration (MF), ultrafiltration (UF), nanofiltration (NF) and reverse osmosis (RO) are based on the principle of selective permeation of solutes through polymeric or inorganic semi-permeable membranes: the driving force for mass transfer of solutes across the membrane is a mechanical pressure. NF is a unit operation which separation characteristics between UF and RO whose molecular weight cut-off (MWCO) ranges from 100 to 1000 Da (g·mol^−1^). It appears to have great potential in the production of high quality food, including water softening, wastewater treatment, beverage industry, dairy industry and sugar industry [23]. The recovery of biologically active compounds from agro-food by-products, also in combination with other membrane operations (*i.e.*, UF and RO), is another research area of growing interest. For example, protein hydrolysates produced by enzymatic hydrolysis of tuna dark muscle, a by-products of tuna processing industry, were fractionated with UF and NF membranes for the production of three different fractions: a fraction rich in peptides of molecular weight (MW) higher than 4 kg/mol; a fraction enriched in peptides with a MW in the range 1–4 kg/mol and a fraction enriched in free amino-acids and dipeptides [24]. Similarly, the fractionation of olive mill wastewaters through the sequential use of UF and NF membranes, produced three different streams: a concentrated fraction enriched in free low molecular weight polyphenols, a concentrate stream enriched in organic substances and a purified water stream which can be reused during the olive oil extraction [25]. In the fractionation of cheese whey, UF and NF membranes were employed in order to obtain valuable components, such as proteins, lactose and minerals, which can be further used in the food industry [26].

In a previous work, a conceptual process design for recovering phenolic compounds and sugars from artichoke wastewaters was proposed on the basis of a NF treatment of clarified artichoke extracts with different NF membranes in selected operating conditions [27].

The current study aimed at validating the proposed flow sheet through the implementation of a two-step NF process with a sequential design in optimized operating conditions. In particular, the clarified artichoke extract was submitted to a first NF process by using a spiral-wound membrane with a MWCO of 400 Da in order to concentrate phenolic compounds in the retentate fraction and to produce a permeate fraction enriched in sugars. The permeate stream was then processed with a NF spiral-wound membrane with a MWCO of 150–300 in order to produce concentrated sugar solutions. For both membranes the effect of the transmembrane pressure (TMP) on permeation fluxes was analyzed. Batch concentration experiments were performed in optimized conditions of TMP. Fractionated streams were characterized in terms of total antioxidant activity (TAA), sugars and phenolic compounds. The performance of selected membranes in terms of productivity and selectivity towards compounds of interest was evaluated and discussed.

## 2. Results and Discussion

### 2.1. Experiments with the NP030 Membrane

The separation of phenolic compounds from sugars in a complex matrix such as the artichoke extract is a difficult task, because the membrane performance does not depend only by membrane characteristics, in particular membrane pore size [28] and membrane material [29], but also by the fouling mechanism produced on the membrane surface and inside membrane pores, which affects the rejection of different compounds. In this regard, NF experiments according to the total recycle configuration with the NP030 membrane were performed at different TMP values in order to optimize the conditions producing maximum differences in the rejection towards phenolic compounds and sugars as well. The physiochemical characteristics of the artichoke extracts submitted to the NF treatment are reported in Table 1.

**Table 1:** Physicochemical composition of the clarified artichoke extract.

Parameters	Permeate
pH	4.1 ± 0.1
TSS (°Brix)	2.4 ± 0.1
Suspended solids (%)	n.d.
Glucose (mg/L)	642.0 ± 3.0
Fructose (mg/L)	577.0 ± 4.0
Sucrose (mg/L)	745.0 ± 1.0
Total polyphenols (mg/L gallic acid)	479.1 ± 2.9
Chlorogenic acid (mg/L)	176.3 ± 2.4
Cynarin (mg/L)	110.6 ± 0.8
Apigenin 7-*O*-glucoside (mg/L)	101.8 ± 0.3
TAA (mM trolox)	5.33 ± 0.05

#### 2.1.1. Experiments according to the Total Recycle Configuration

**Figure 1:**
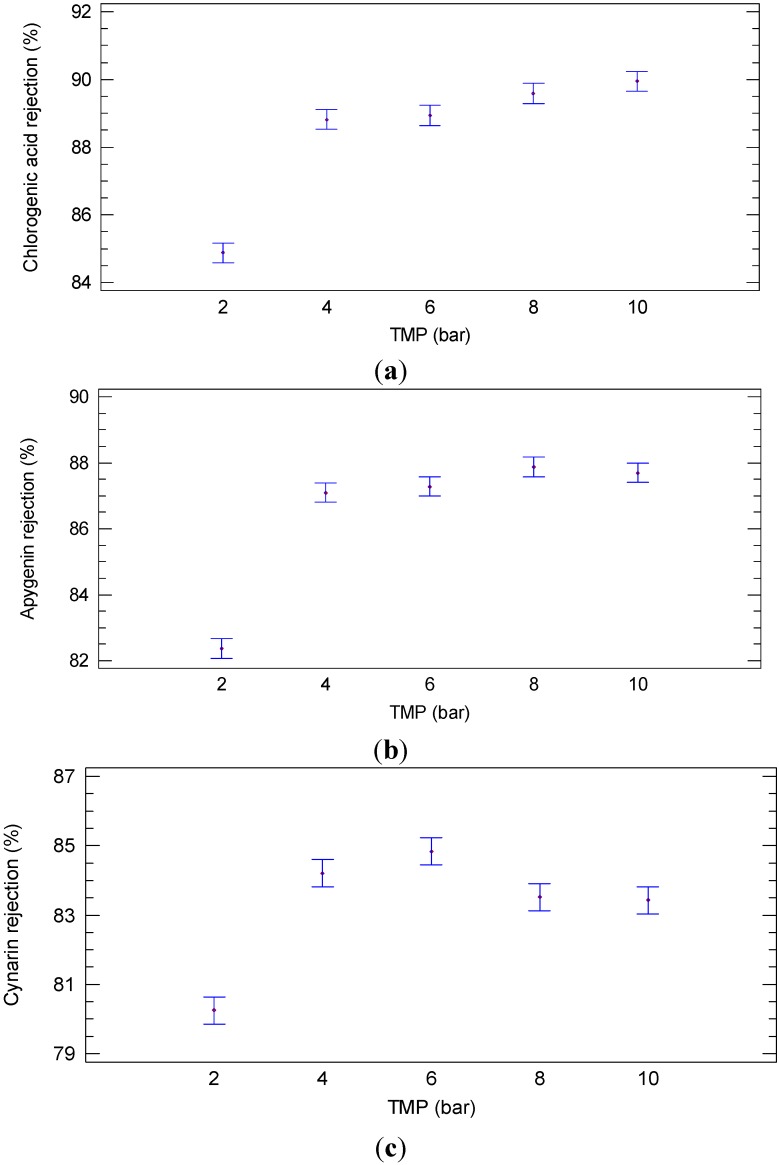
Least significant difference (LSD) plot showing the effect of transmembrane pressure (TMP) on the rejection of (**a**) chlorogenic acid (**b**) apygenin and (**c**) cynarin (NP030 membrane, total recycle configuration, 25 °C).

Figure 1 shows the rejection of the NP030 membrane towards chlorogenic acid, cynarin and apygenin in the range of TMP values of 2–10 bar. The rejection towards chlorogenic acid shows a significant difference (*p* = 0.000; F-ratio = 119.31) at different TMP values. The minimum rejection was observed at 2 bar, whereas the maximum rejection was obtained at 10 bar. It was appreciated that there is no significant difference in the rejection between 4 and 6 bar as well as between 8 and 10 bar (Figure 1a). Similar results were obtained for apygenin where significant differences (*p* = 0.000; F-ratio = 155.20) were observed in the rejection at different TMP values. The minimum rejection was obtained at 2 bar and the maximum one at 8 bar. According to LSD analysis there are no significant differences between 4–6 and 8–10 bar (Figure 1b). On the other hand, significant differences (*p* = 0.000; F-ratio = 51.66) were found in the rejection of cynarin at the TMP values investigated. In this case, the minimum rejection was found at 2 bar such as for chlorogenic acid and apygenin; however the maximum rejection was at 4–6 bar (Figure 1c).

**Figure 2:**
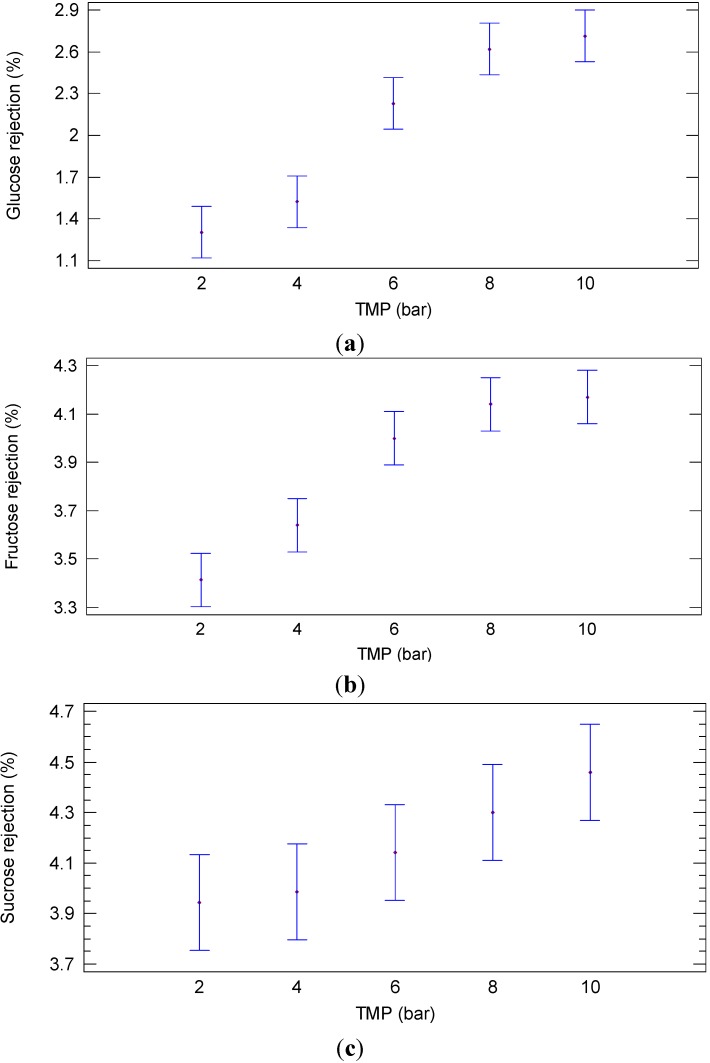
Least significant difference (LSD) plot showing the effect of TMP on the rejection of (**a**) glucose (**b**) fructose and (**c**) sucrose (NP030 membrane, total recycle configuration, 25 °C).

A different behavior was observed in relation to the rejection of the selected membrane towards sugar compounds. The rejection of the NP030 membrane towards sugars at different TMP values is illustrated in Figure 2. Significant differences (*p* = 0.000; F-ratio = 43.68) were found between the sugar rejection at different TMP values. In particular, low rejection values towards glucose (Figure 2a), fructose (Figure 2b) and sucrose (Figure 2c) were observed at 2–4 bar when compared with higher operating pressures (10 bar).

**Figure 3:**
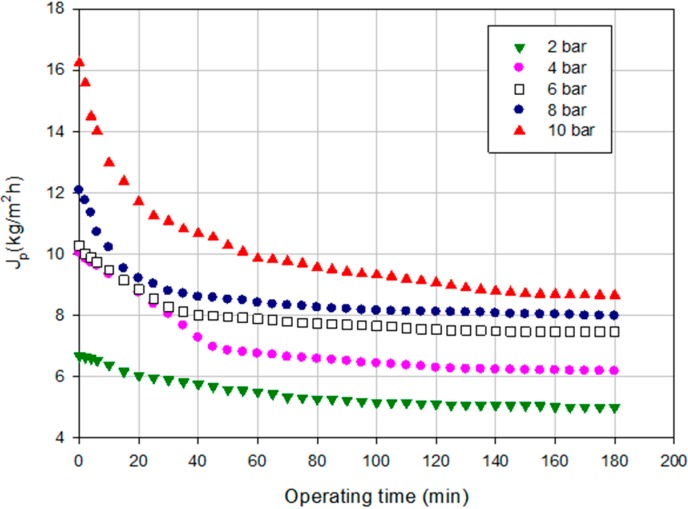
Time course of permeate flux at different TMP values (NP030 membrane, total recycle configuration, 25 °C).

Figure 3 shows the time evolution of the permeate flux in the investigated range of operating pressures. The increase in TMP produced an increasing in the permeate flux; in addition, the flux decay increased by increasing the TMP. In particular, at 10 bar, the drop in the permeate flux was of 46% when compared with the membrane performance at 2 bar where the drop in the permeate flux was of 25%. This behavior can be attributed to the concentration polarization and membrane fouling produced on the membrane surface [30].

**Figure 4:**
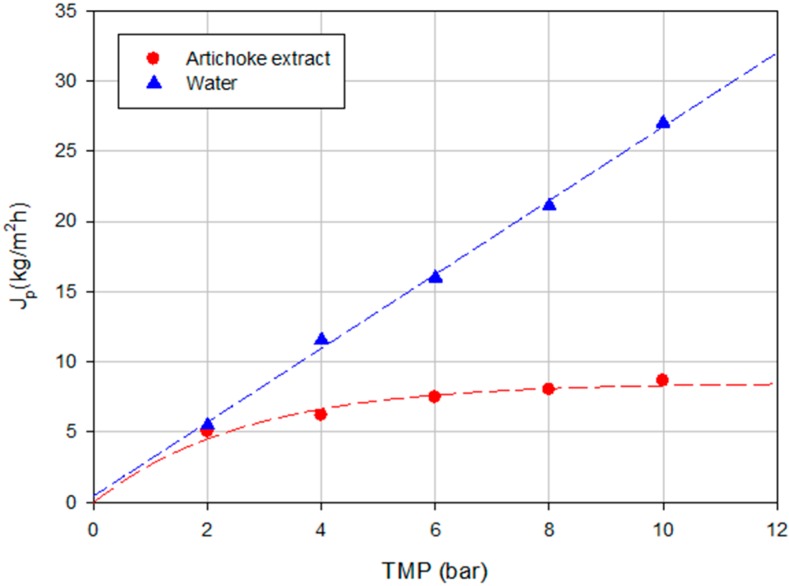
Influence of TMP on the permeate flux of water and the clarified artichoke extract (NP030 membrane, total recycle configuration, 25 °C).

Figure 4 shows the steady-state permeate flux as a function of TMP for the feed solution and water as well. Field *et al.* [31] described two forms of critical flux; a strong form and a weak form. The strong form of the critical flux exists when the flux up to a point is equivalent to the corresponding pure water flux at the same pressure. For the weak form, it is assumed that there is a very rapid fouling on start-up and so the flux-TMP relationship is below that of the pure water line. The critical flux (weak form) is the point at which this line becomes non-linear [32]. According to Figure 4, after two bars it is observed that the line becomes non-linear indicating the presence of fouling on the membrane surface. Fouling phenomena produce not only a drop in the permeate flux but also an increase in the rejection towards phenolic compounds. Therefore, in this case, the fouling phenomena can contribute to achieve the expected results in terms of high rejection towards phenolic compounds.

The results obtained according to a total recycle configuration were useful to select the TMP conditions for the experiences performed under the batch concentration mode. In this step the objective was focused to obtain the maximum separation between polyphenols and sugars. In this regard, the minimum rejection towards sugars was obtained at 4 bar, whereas the rejection of polyphenols was higher than 83% for all the phenolic compounds measured.

#### 2.1.2. Experiments according to the Batch Concentration Configuration

**Figure 5:**
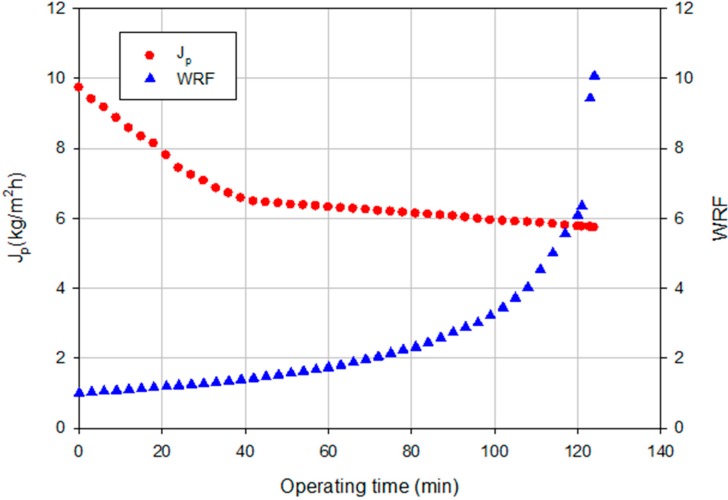
Nanofiltration (NF) of clarified artichoke extract with NP030 membrane. Time course of permeate flux and weight reduction factor (WRF) (batch concentration configuration, 4 bar, 25 °C).

Figure 5 shows the time evolution of the permeate flux for the selected membrane at an operating TMP of 4 bar according to a batch concentration configuration up to a final weight reduction factor (WRF) of 10. The permeate flux drop was equal to 40.8% with a pseudo-stable flux of 5.86 kg/m^2^·h due to fouling phenomena.

**Figure 6:**
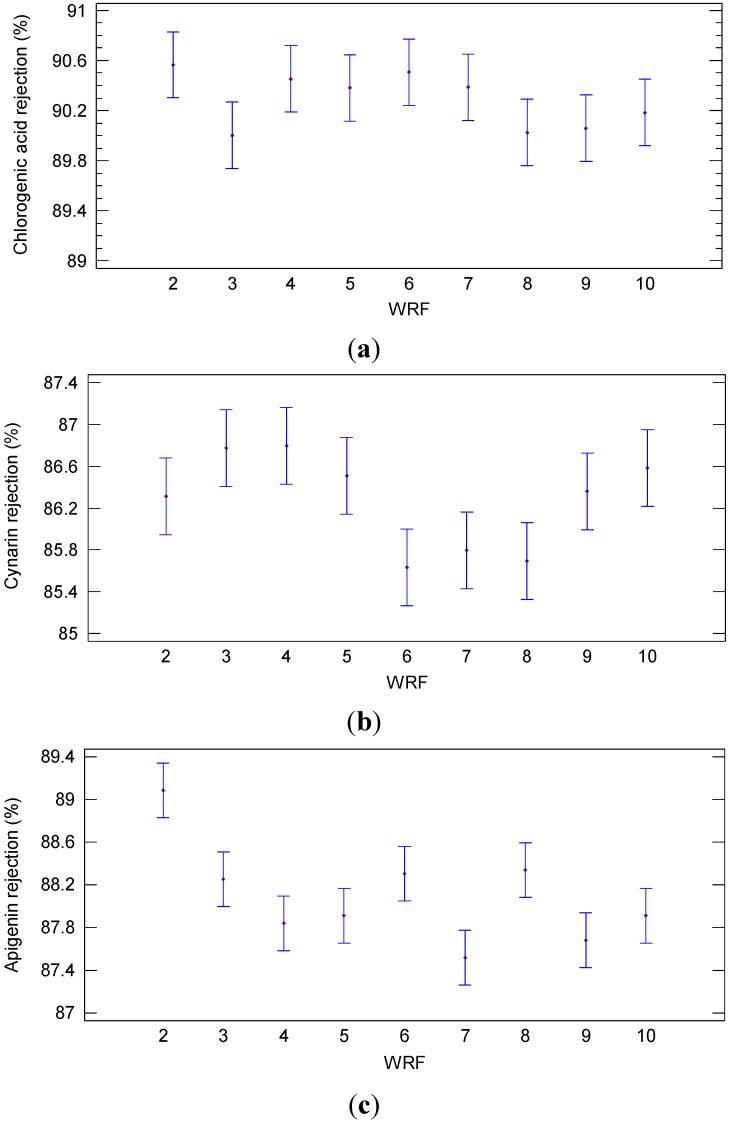
Least significant difference (LSD) plot showing the effect of WRF on the rejection of (**a**) chlorogenic acid (**b**) cynarin and (**c**) apigenin (NP030 membrane, batch concentration configuration, 4 bar, 25 °C).

In these conditions, the rejection towards chlorogenic acid, cynarin and apigenin was higher than 85% independently of the achieved WRF as shown in Figure 6. In particular, the rejection towards chlorogenic acid did not show significant differences (*p* = 0.2202; F-ratio = 1.52) at WRF from 2 to 10 (Figure 6a). On the other hand, significant differences (*p* = 0.144; F-ratio = 3.42) were observed for the cynarin rejection at different WRF (Figure 6b). The same behavior was observed for apigenin where the rejection showed significant differences (*p* = 0.002; F-ratio = 7.41) from WRF 2 to 10 (Figure 6c).

For these compounds the increasing of the WRF did not produce a significant increasing of the rejection towards phenolic compounds. Therefore, the increasing in feed concentration did not affect the fouling phenomena. At the highest WRF, in the selected operating conditions, a retentate stream with concentrations of chlorogenic acid, cynarin and apigenin of 1224, 898 and 814 ppm, respectively, was obtained. On the other hand, the concentration of these compounds in the permeate stream was 10 times lower than the initial feed and not affected by the WRF (Table 2).

**Table 2:** Effect of WRF on the phenolic composition of permeate and retentate streams from clarified artichoke extract treated with NP030 membrane.

Sample	WRF	Chlorogenic Acid (mg/L)	Cynarin (mg/L)	Apigenin (mg/L)
Feed	-	168.00 ± 0.55	103.00 ± 0.60	96.20 ± 0.60
Permeate	2	15.85 ± 0.63	14.10 ± 0.50	10.50 ± 0.30
	3	16.80 ± 0.84	13.62 ± 0.17	11.30 ± 0.10
	4	16.04 ± 0.76	13.60 ± 0.90	11.70 ± 0.10
	5	16.17 ± 0.38	13.90 ± 0.70	11.65 ± 0.17
	6	15.95 ± 0.35	14.80 ± 0.60	11.25 ± 0.45
	7	16.15 ± 0.43	14.63 ± 0.21	12.01 ± 0.39
	8	16.76 ± 0.62	14.74 ± 0.62	11.22 ± 0.68
	9	16.70 ± 0.35	14.05 ± 0.80	11.85 ± 0.25
	10	16.49 ± 0.53	13.82 ± 0.37	11.65 ± 0.25
Retentate	2	201.70 ± 2.40	172.90 ± 2.70	146.40 ± 1.90
	3	257.15 ± 1.62	213.4 ± 1.10	198.10 ± 0.50
	4	347.10 ± 1.55	304.45 ± 1.95	259.45 ± 0.85
	5	452.60 ± 0.84	414.4 ± 1.80	347.25 ± 1.05
	6	512.70 ± 0.98	482.90 ± 2.70	414.55 ± 2.05
	7	603.25 ± 1.34	577.25 ± 0.95	505.25 ± 1.00
	8	702.80 ± 0.56	682.80 ± 2.60	647.05 ± 0.25
	9	917.30 ± 1.55	766.90 ± 1.50	703.85 ± 0.45
	10	1224.00 ± 2.19	898.05 ± 0.55	814.35 ± 2.05

**Figure 7:**
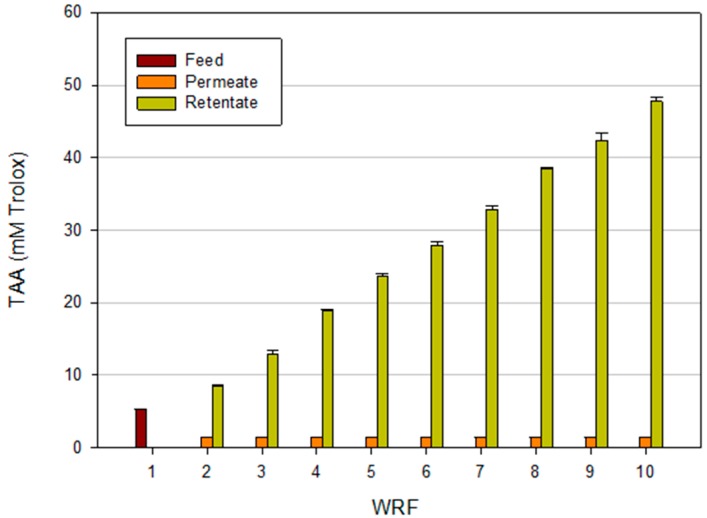
TAA as a function of WRF for permeate and retentate streams of clarified artichoke extracts treated with NP030 membrane.

The increased concentration of phenolic compounds in the retentate was accompanied by an increasing of the total antioxidant activity (TAA) stream as reported in Figure 7. In particular, the ABTS radical scavenging capacity of retentates was increased by 9 times in comparison with the initial feed. Similar results were obtained in the concentration of phenolic compounds from mate bark aqueous extracts [33] and from ethanolic extracts of *Sideritis* ssp. (an endemic plant of the Balkan Peninsula) [34] by NF membranes. At the same time, the TAA of the permeate remained low and unchanged in the permeate stream. These results confirmed the strict correlation between total phenolic content and antiradical activity widely reported in literature [35,36,37].

**Figure 8:**
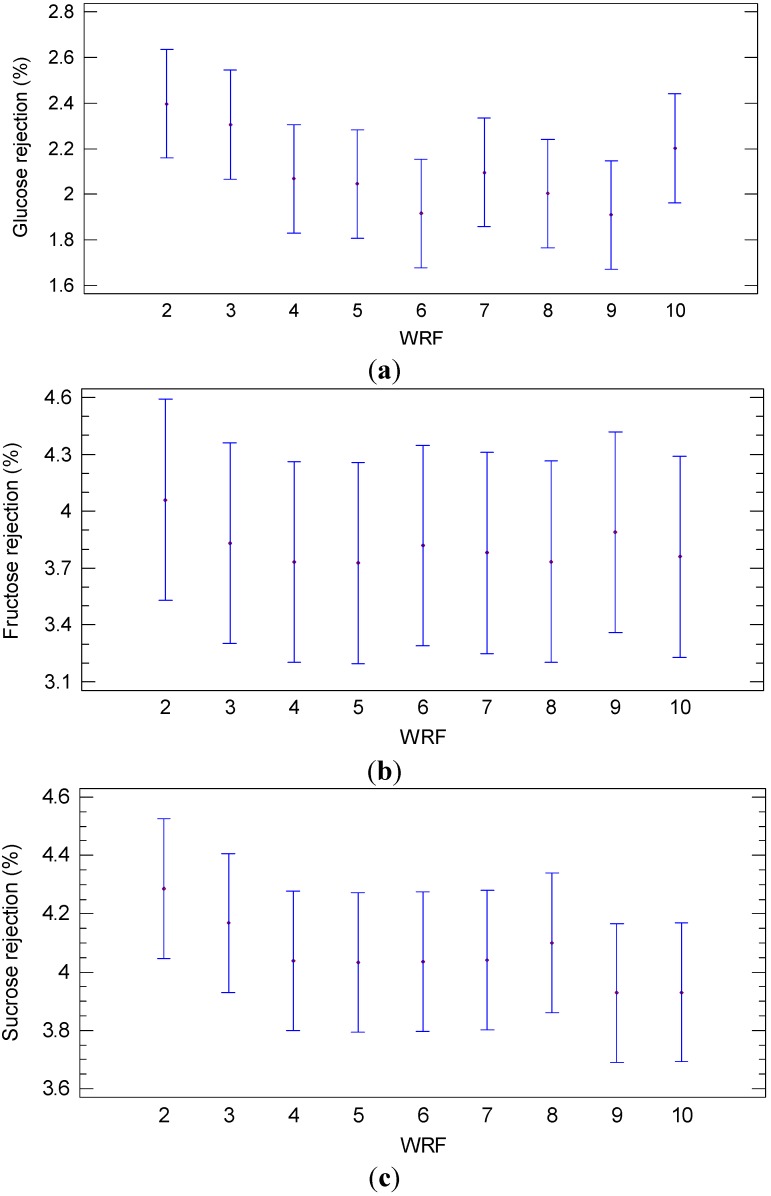
Least significant difference (LSD) plot showing the effect of WRF on the rejection of (**a**) glucose (**b**) fructose and (**c**) sucrose (NP030 membrane, batch concentration configuration, 4 bar, 25 °C).

Rejections of the NP030 membrane towards glucose, fructose and sucrose measured at different WRF in the same operating conditions are illustrated in Figure 8. In particular, no significant differences (*p* = 0.4152; F-ratio = 1.09) were observed in the rejection towards glucose at different WRF (Figure 8a). Similar results were observed for fructose, where the differences were not significant (*p* = 0.9992; F-ratio = 0.09) (Figure 8b), and sucrose (*p* = 0.8512; F-ratio = 0.48) as well (Figure 8c). According to these results, the low rejection towards sugars (lower than 4.8%) allowed to obtain a permeate stream with high concentrations of glucose, fructose and sucrose (617, 547 and 708 ppm, respectively). This permeate stream was used as a feed solution in the next NF step.

### 2.2. Experiments with the Desal DK Membrane

The permeate stream obtained in the first NF step was used as a feed solution in the second NF step performed with the Desal DK membrane. The chemical composition of this solution is reported in Table 3.

**Table 3:** Chemical composition of the feed solution for Desal DK membrane.

Parameters	Feed
Glucose (mg/L)	617.15 ± 0.43
Fructose (mg/L)	547.75 ± 0.70
Sucrose (mg/L)	708.70 ± 0.70
Chlorogenic acid (mg/L)	16.49 ± 0.53
Cynarin (mg/L)	13.82 ± 0.38
Apigenin 7-*O*-glucoside (mg/L)	11.62 ± 0.22
TAA (mM trolox)	1.39 ± 0.01

#### 2.2.1. Experiments according to the Total Recycle Configuration

**Figure 9:**
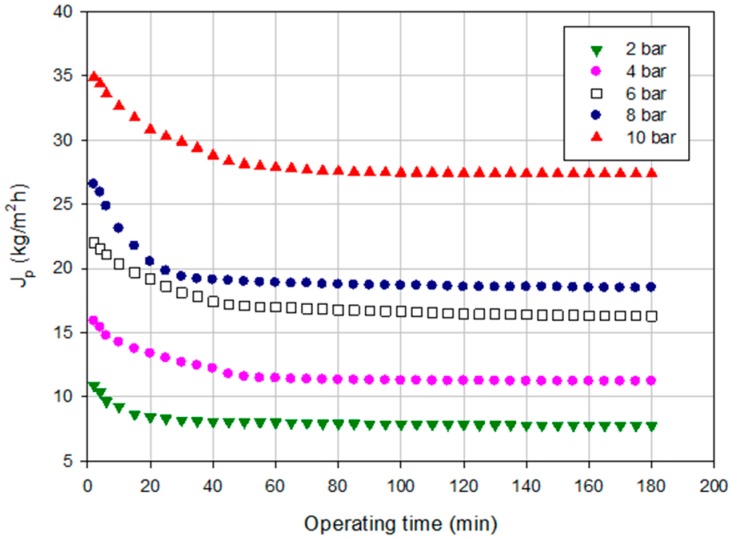
Time course of permeate flux at different TMP values (Desal DK membrane, total recycle configuration, 25 °C).

**Figure 10:**
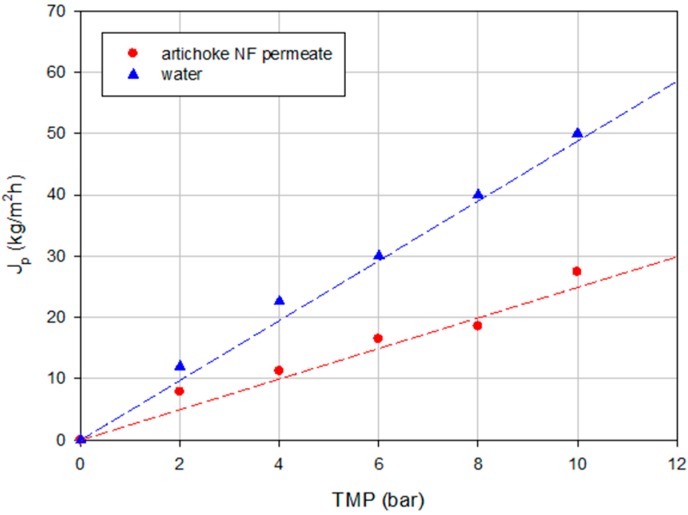
Influence of TMP on the permeate flux of water and the NF permeate of the artichoke extract (Desal DK membrane, total recycle configuration, 25 °C).

The effect of TMP on the rejection towards sugars as well as the behavior of permeate flux at different TMP was studied. Figure 9 shows the time evolution of the permeate flux at different TMP values according to a total recycle configuration for the Desal DK membrane. Significant differences (*p* = 0.0000; F-ratio = 868.77) were found between the permeate flux values within the operating pressures investigated. Figure 10 shows the relationship between TMP and steady-state permeate flux for the artichoke extract and water. In both cases a linear trend can be observed. The difference between the permeate fluxes of water and artichoke extract shows the amount of fouling in the process under the same conditions of temperature and pressure. A linear trend between permeate flux and pressure was also observed by Mello *et al.* [38] in the concentration of phenolic compounds in aqueous and ethanolic propolis extracts through a NF membrane. According to Bacchin *et al.* [32] this behavior represents a presence of not complete adsorption and possible mass deposition by convection. In our case, a critical flux is not reached at all and also at the maximum TMP investigated the linearity between TMP and permeate flux is maintained. Therefore, operating at 10 bar it will be possible to obtain permeate flux values below the region where the fouling becomes irreversible.

The analyses of sugar compounds in the permeate stream revealed that these compounds were completely retained by the selected membrane.

#### 2.2.2. Experiments according to the Batch Concentration Configuration

**Figure 11:**
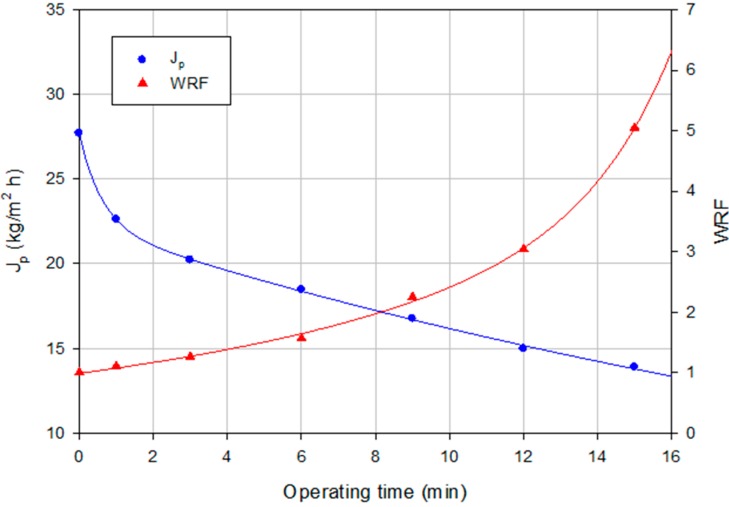
Nanofiltration of NP030 permeate stream with the Desal DK membrane. Time course of permeate flux and WRF (batch concentration configuration, 10 bar, 25 °C).

Experimental runs according to the batch concentration configuration were performed in optimal operating conditions on the basis of the results obtained under a total recycle configuration. Figure 11 shows the time evolution of the permeate flux and WRF when the NF process was operated at a TMP of 10 bar and a temperature of 25 °C up to a final WRF of 5. In this case, the reduction of permeate flux was equal to 49.83% achieving a pseudo-stable permeate flux of 13.89 kg/m^2^·h.

Table 4 shows the chemical composition in terms of phenolic compounds and sugars of permeate and retentate fractions collected at different WRF values in the experiments performed according to the batch concentration configuration. As it can be seen, both phenolics and sugars were not detectable in the permeate. Their concentration in the retentate streams increased by increasing the WRF and the concentration factor in the retentate collected at WRF 5 was in agreement with the complete retention of these compounds. However, being most part of phenolic compounds removed in the first NF step, the concentration of sugar compounds in the final retentate resulted much higher than that of phenolic compounds. In particular, the initial ratio between sugar and phenolic concentration in the aqueous extract was increased by about 10 times in the final retentate.

**Table 4:** Analyses of sugars and phenolic compounds in samples coming from the treatment of the NP030 permeate stream with the Desal DK membrane.

Sample	WRF	Sugars			Phenolic Compounds		
		Glucose (mg/L)	Fructose (mg/L)	Sucrose (mg/L)	Chlorogenic Acid (mg/L)	Cynarin (mg/L)	Apigenin-7-*O*-Glucoside (mg/L)
Feed	-	631.0 ± 2.2	569.1 ± 3.1	737.7 ± 1.7	14.5 ± 0.3	12.5 ± 0.1	9.6 ± 0.1
Permeate	2–5	n.d.	n.d.	n.d.	n.d.	n.d.	n.d.
Retentate	2	903.0 ± 1.4	815.0 ± 1.3	1113.5 ± 1.0	22.5 ± 0.3	17.5 ± 1.1	14.6 ± 0.2
	3	1521.0 ± 0.1	1235.5 ± 1.0	1814.5 ± 1.9	36.1 ± 0.7	27.7 ± 0.5	21.8 ± 0.5
	4	2094.7 ± 4.6	1814.4 ± 2.0	2613.5 ± 1.2	45.5 ± 1.0	37.5 ± 0.9	30.4 ± 0.2
	5	2715.2 ± 1.5	2221.9 ± 1.6	3113.8 ± 1.0	67.5 ± 1.2	55.5 ± 1.3	43.6 ± 1.0

## 3. Experimental Section

### 3.1. Feed Solution

The plant extract investigated here was prepared by using artichoke hearts (of Italian variety) purchased at a local market. Artichokes were crushed and mixed with deionized water (in a proportion of 40 kg in 100 L) to be cooked at 85 °C for 30 min. Then, residues of artichoke were separated from the solution by a filtration with nylon cloth. The aqueous extract can be assimilated to the waste product obtained in the blanching step during the industrial transformation of artichokes. Blanching is a short heat treatment aimed to inactivate enzymes associated to the enzymatic browning (such as polyphenol oxidase and peroxidase) and to obtain a partial cooking of the artichokes before freezing, canning or fermentation. The solution was previously clarified by ultrafiltration (UF). The clarification was carried out by using a laboratory unit supplied by Verind SpA (Milan, Italy) equipped with a polysulphone membrane module (DCQ III-006C, China Blue Star Membrane Technology Co., Ltd., Beijing, China) in hollow fiber configuration with an effective membrane area of 1.2 m^2^. The UF process was operated at a transmembrane pressure (TMP) of 0.3 ± 0.01 bar, a temperature of 21 ± 1 °C and a feed flow rate of 550 L/h according to a batch concentration mode (the retentate stream was recycled back to the feed reservoir and the permeate was collected separately). The clarified wastewater was stored at −17 °C and defrosted to room temperature before NF experiments.

### 3.2. NF Equipment and Procedures

NF experiments with artichoke extracts were performed by using a laboratory plant supplied by Matrix Desalination Inc. (Fort Lauderdale, FL, USA). The equipment consists of a feed tank with a capacity of 12 liters, a stainless steel housing for 2.4 × 21 inches spiral-wound membrane module, a high pressure pump, a back-pressure valve, two pressure gauges, a permeate flowmeter and a permeate tank. A cooling coil, fed with tap water, was used in the feed tank to control the feed temperature.

In the first step, clarified artichoke extracts were processed by using a polyethersulphone NF spiral-wound membrane module (NP030, Microdyn Nadir, Wiesbaden, Germany) with a nominal molecular weight cut-off (NMWCO) of 400 Da and an effective membrane area of 1.8 m^2^. Experiments were performed in both total recycle configuration (recycling both permeate and retentate streams in the feed tank of the plant) and batch concentration configuration. In the former TMP was varied from 2 to 10 bar at a selected temperature of 25 °C. The clarified artichoke extract was processed according to a batch concentration configuration at a TMP of 4 bar and a temperature of 25 °C up to reach a final weight reduction factor (WRF) of 10.

In the second step, the NF permeate was processed with a spiral-wound cross-linked aromatic polyamide Desal DK2540 membrane supplied by GE Water & Process Technologies (Trevose, PA, USA) with a NMWCO of 150–300 Da and an effective membrane area of 2.6 m^2^. The TMP in the experiences performed according to a total recycle configuration was varied from 2 to 10 bar at a selected temperature of 25 °C. The NF permeate was processed also according to a batch concentration configuration at a TMP of 10 bar and a temperature of 25 °C up to a final WRF of 5.

The permeate flux (*J*) was determined by measuring the collected permeate weight in a given time through the membrane surface area by using the following equation:

(1) $$J_{p}=\frac{W_{p}}{At}$$

where *W_p_* is the weight of collected permeate through the membrane surface area of permeation *A*, in a time *t*.

The rejection of NF membranes towards specific compounds was calculated as:

(2) $$R=\left(1-\frac{C_{p}}{C_{f}}\right)\times 100$$

where *R* is the membrane rejection while *C_p_* and *C_f_* represent the concentration of a specific compound in the permeate stream and in the feed solution.

### 3.3. Analytical Methods

#### 3.3.1. Total Phenols

Total phenols were estimated colorimetrically by using the Folin–Ciocalteu method [39]. The method is based on the reduction of tungstate and/or molybdate in the Folin-Ciocalteu reagent by phenols in alkaline medium resulting in a blue colored product (λ_max_ 756 nm). The estimation of total phenols was carried out in triplicate and results were expressed as mg/L gallic acid.

#### 3.3.2. Polyphenols

Polyphenols were assessed by using an HPLC system (Agilent 1100 Series, Santa Clara, CA, USA) equipped with a pump, a UV-Vis detector and a data acquisition system. Chromatographic separation was performed by using a Luna C 18(2) column (250 × 4.6 mm, 5 μm, Phenomenex, Torrance, CA, USA).

The following conditions were used: *V* = 1 mL/min; *T* = 25 °C; λ = 320 nm. The mobile phase was a mixture of H_2_O/HCOOH (9:1) as solvent A and H_2_O/HCOOH/CH_3_CN (4/1/5) as solvent B. Polyphenols separation was achieved by using the following linear gradient: starting condition, 90% A, 10% B; 30 min, 50% A, 50% B; 35 min, 100% B; 35 min, 0% A, 100% B. Analyses were stopped after 56 min. Polyphenols were identified by matching the retention time and their spectral characteristics against those of standards (chlorogenic acid, cynarin, apigenin 7-*O*-glucoside). Quantification was made according to the linear calibration curves of standard compounds.

#### 3.3.3. Sugars

The quantitative determination of glucose and sucrose was carried out by using an HPLC system (Thermo Scientific Accella 600, San Jose, CA, USA) equipped with a binary pump, an autosampler, a thermostated column compartment and a refractometer index detector.

Separation was achieved on Phenomenex Luna 5u·NH_2_ 100A column (250 mm × 4.60 mm, 5 μm, Phenomenex, Torrance, CA, USA). Samples were eluted in isocratic mode by using a mixture of ACN/H_2_O (8:2). Operating conditions were as follows: *V* = 1 mL/min, *T* = 40 °C, pressure = 85 bar.

Prior to HPLC analysis all samples were diluted with ACN (4:1) and filtered by using 0.45 μm nylon filters. A sample volume of 20 μL was used.

#### 3.3.4. Total Antioxidant Activity (TAA)

TAA was determined by an improved version of the ABTS radical cation decolorization assay [40,41] in which the radical cation 2,2-azino-*bis*-(3-ethylbenzothiazoline-6-sulphonic acid) (ABTS^+^) is generated by reaction with potassium persulfate before the addition of the antioxidant. This method gives a measure of the antioxidant activity of pure substances and of mixtures by monitoring the reduction of the radical cation as the percentage inhibition of absorbance at 734 nm. Spectrophotometric measurements were performed by using a UV-Visible recording spectrophotometer (UV-160 A, Shimadzu Scientific Instruments, Inc., Kyoto, Japan) at 30 °C. ABTS was dissolved in water at 2 mM concentration: ABTS radical cation was produced by reacting 10 mL of ABTS stock solution with 100 μL of 70 mM potassium persulfate solution (ABTS:K_2_S2O_8_ = 1:0.35 molar ratio) and allowing the mixture to stand in the dark at room temperature for 12–16 h before use. Work solution was prepared diluting 1 mL of the ABTS radical cation solution to 25 mL with PBS buffer (5 mM·Na_2_HPO_4_, 5 mM·NaH_2_PO_4_, NaCl 9 g/L, pH = 6.8) to a final UV absorbance of 0.70 ± 0.02 at 734 nm. After addition of 10 μL of sample to 10 mL of ABTS work solution, the absorbance at 734 nm was recorded every min for a total of 6 min. The value at 5 min was used to calculate the results reported as TAA, expressed in terms of mM trolox equivalent. Each determination was performed in triplicate. Results were expressed as mean ± SD of three samples.

### 3.4. Data Analysis

Least significant difference (LSD) analysis was used to compare the results obtained in terms of permeate flux and rejection towards phenolic compounds and sugars.

## 4. Conclusions

A two-step NF process was investigated in order to purify phenolic compounds from an aqueous artichoke extract. Two spiral-wound NF membranes were selected and used in a sequential design to treat the artichoke extract previously clarified by UF.

For both membranes optimal operating pressures were identified in experiments performed according to the total recycle configuration.

In optimized operating conditions (4 bar, 25 °C) most part of phenolic compounds were retained by the first NF membrane (NP030) while sugar compounds were recovered in the permeate stream. The rejection towards chlorogenic acid, cynarin and apigenin resulted higher than 85% independently of the achieved WRF. A pseudo-stable permeate flux of 5.86 kg/m^2^·h was measured when the NF process was operated according to a batch concentration configuration.

The permeate stream obtained in the first NF step was used as a feed solution in the second NF step performed with the Desal DK membrane. Sugar compounds were totally retained by the NF membrane when the process was operated at 10 bar and 25 °C under batch concentration configuration. The permeate stream was a clear solution free of sugars and phenolic compounds. A permeate flux of about 15 kg/m^2^·h was measured in the selected operating conditions at a WRF of 5.

According to the investigated process the aqueous artichoke extract can be fractionated in three different valuable streams:

- a retentate fraction from the first NF step enriched in phenolic compounds with high antioxidant activity (the ABTS radical scavenging capacity was increased by 9 times in comparison with the initial feed) of interest for nutraceutical or food applications;
- a retentate fraction from the second NF step enriched in sugar compounds;
- a clear permeate from the second NF step free of sugars and phenolic compounds which can be reused for irrigation or recycled in the artichoke processing industry.
